# The evidence to date: implications of l-ascorbic acid in the pathophysiology of aging

**DOI:** 10.1186/s12576-024-00922-7

**Published:** 2024-05-11

**Authors:** Ayami Sato, Yoshitaka Kondo, Akihito Ishigami

**Affiliations:** 1 Molecular Regulation of Aging, Tokyo Metropolitan Institute for Geriatrics and Gerontology, Tokyo, 173-0015 Japan; 2 Department of Nutritional Sciences, Faculty of Health and Sports Sciences, Toyo University, Tokyo, 115-8650 Japan

**Keywords:** L-ascorbic acid, Vitamin C, Vitamin C-deficient models, Lifespan, Age-related diseases

## Abstract

L-Ascorbic acid, commonly known as vitamin C, has been used not only for disease prevention and in complementary and alternative medicine, but also for anti-aging purposes. However, the scientific evidence is not yet sufficient. Here, we review the physiological functions of vitamin C and its relationship with various pathological conditions, including our previous findings, and discuss the prospects of its application in healthy longevity. In summary, vitamin C levels are associated with lifespan in several animal models. Furthermore, clinical studies have shown that the blood vitamin C levels are lower in middle-aged and older adults than in younger adults. Lower blood vitamin C levels have also been observed in various pathological conditions such as chronic kidney disease and chronic obstructive pulmonary disease in the elderly. These observations suggest the implications of vitamin C in age-related pathological mechanisms owing to its physiological functions.

## Background

Among the members of the vitamin family, l-ascorbic acid (vitamin C) is particularly well known to the public, and functional foods and supplements are widely available. Vitamin C is a water-soluble antioxidant that was discovered as an anti-scurvy factor. Therefore, long-term vitamin C deficiency can cause scurvy. The main symptoms of scurvy include gingival hemorrhage, arthralgia, impaired wound healing, perifollicular hemorrhage, and ecchymoses [1]. Blood vitamin C levels of healthy individuals are around 50 µM, and levels below 11 µM increase the risk of scurvy [2]. Under physiological pH conditions in aqueous solution, vitamin C most commonly exists in its monoanionic form, ascorbate. Dehydroascorbic acid (DHA), an oxidized form of ascorbate, is generated via redox reactions [3]. In contrast, in vivo, DHA is reduced to ascorbate by dehydroascorbate reductase. In this article, ascorbate is described as vitamin C and L-ascorbic acid.

A reference value of 95–110 mg/day for vitamin C intake is recommended for healthy adults to maintain an adequate status [4]. There have been concerns regarding the accumulation and deposition of a vitamin C metabolite, oxalate, in the kidneys with increased intake of vitamin C [5]. However, recent examinations indicate that excess vitamin C is not associated with urinary stones or kidney injury [6]. Thus, vitamin C has few adverse effects, and high-dose therapy has been used worldwide. However, its efficacy remains unclear.

Notably, lower blood vitamin C levels were observed in various age-related pathological conditions [7, 8]. These manifestations are thought to be closely related to lower blood vitamin C levels in middle- and older-aged adults. Nevertheless, previous reports on vitamin C have not been summarized for some age-related diseases, but only for specific diseases. In this article, we review the physiological functions of vitamin C and its relationship with various pathological conditions, and discuss the implications of vitamin C in age-related diseases, including our previous findings.

## Main text

### Physiological functions of vitamin C

Vitamin C is a scavenger of reactive oxygen species (ROS) and an important cofactor in enzymatic reactions [9]. The enzymatic reactions catalyzed in the presence of vitamin C are categorized two main types, 2-oxoglutarate (α-ketoglutarate) dependent (or -independent) iron (Fe^2+^)-containing dioxygenases and copper (Cu^2+^)-containing monooxygenases. In Fe^2+^-containing dioxygenases reactions, vitamin C acts on hydroxylation and is involved in collagen polymerization, tyrosine metabolism, proteolysis of hypoxia-inducible factor (HIFα), and epigenetic regulations such as DNA, RNA, and histone demethylation [10]. In contrast, Cu^2+^-containing monooxygenase reactions with vitamin C catalyze the synthesis of peptide hormones and catecholamines [9]. These properties of vitamin C are thought to maintain biological functions and lead to anti-inflammatory and immune-boosting effects. Vitamin C is also believed to act as a cofactor in the Fe^2+^-containing dioxygenase reactions involved in carnitine synthesis [11]. However, we demonstrated that vitamin C is not essential for carnitine biosynthesis in vivo and suggested that glutathione (GSH) may compensate for vitamin C in this pathway [12].

Furthermore, vitamin C promotes iron absorption [13]. Vitamin C increases the absorption efficiency in the intestinal tract by reducing Fe^3+^ to Fe^2+^ in food.

In addition, vitamin C is known to recycle vitamin E (α-tocopherol) [14]. Vitamin E prevents lipid peroxidation by reducing lipid peroxyl radicals [15]. In contrast, vitamin C restores the antioxidant capacity of vitamin E by donating hydrogen atoms to the vitamin E radicals. Subsequently, oxidized vitamin C is regenerated by GSH in vivo [16]. Previously, we suggested that the interaction between vitamin C and vitamin E is tissue-specific and demonstrated that vitamin C spares vitamin E levels mainly in the liver in a mouse model [17].

Thus, vitamin C functions extensively in biological reactions.

### Studies on vitamin C and lifespan

Many vertebrates synthesize vitamin C in vivo. However, primates, including humans, lack the ability to synthesize vitamin C due to mutations in the L-gulono-γ-lactone oxidase (*Gulo*) gene, the final enzyme in the pathway that biosynthesizes vitamin C from glucose [18]. Therefore, humans must consume external vitamin C to prevent scurvy. In contrast, mice and rats, which are commonly used models for pharmacological experiments, do not harbor mutations in *Gulo*. Therefore, *Gulo*-knockout (KO) mice [19], senescence marker protein-30/gluconolactonase (SMP30/GNL)-KO mice [20], osteogenic disorder Shionogi (ODS) rats [21], and guinea pigs [22] are used as rodent models deficient in vitamin C synthesis. Interestingly, studies on *Gulo*-KO and SMP30/GNL-KO mice have shown that feeding small amounts of vitamin C, which does not cause scurvy, and continued breeding under conditions of vitamin C shortage, shortens their lifespan [23, 24]. SMP30 was found as a protein that decreases with aging in the liver and kidneys of mice and rats [25]. Subsequently, SMP30 was identified as GNL, an enzyme involved in the vitamin C biosynthesis pathway [20]. Our previous studies have shown that vitamin C deficiency or insufficiency leads to age-related disease-like symptoms (e.g., hearing loss [26], UV-induced cataracts [27], epidermal atrophy [28], and impaired physical function [29, 30]) in SMP30/GNL-KO mice. Furthermore, chronic vitamin C deficiency in ODS rats results in senile disease-like lesions such as osteoporosis and emphysema [31].

In *Caenorhabditis elegans*, the efficacy of vitamin C administration in extending lifespan has been shown [32], but other studies have reported no lifespan extension by vitamin C because it acts as a pro-oxidant [33]. In *Drosophila*, no effect of vitamin C administration on lifespan has been observed [34].

In addition, long-term administration of vitamin C to wild-type mice, which have the ability to synthesize vitamin C, did not alter their lifespan compared to unadministered mice [35]. However, the lifespan was shortened in a mouse model of Werner's syndrome, a disease of premature aging, compared to that in wild-type mice, whereas vitamin C administration resulted in a lifespan equivalent to that of wild-type mice [35].

Vitamin C has been shown to exhibit anti-aging properties in several animal models. In contrast, vitamin C supplementation had no effect on mortality in a large human study [36]. However, various confounding factors may have influenced the results. Recently, a positive correlation between vitamin C intake and telomere length has been reported [37]. The new physiological effects of vitamin C are expected to be elucidated in the future. Furthermore, a simple sensing methodology for the rapid detection of vitamin C has been developed [38], and vitamin C levels should be easily monitored to diagnose health.

### Vitamin C and pathologies

**Fig. 1:**
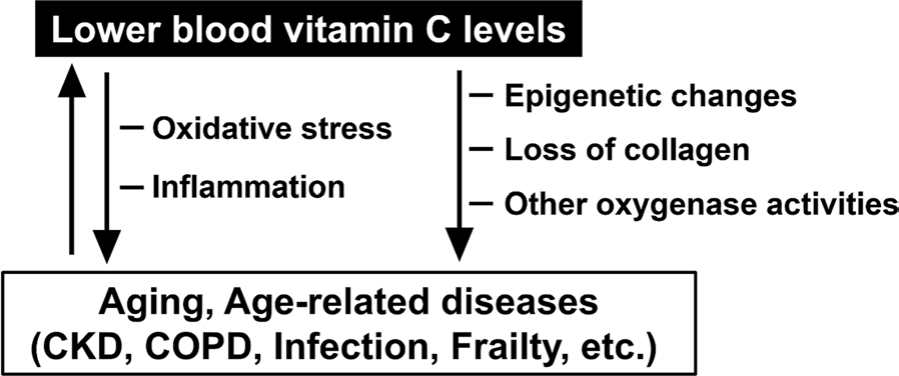
Blood vitamin C level and its physiological contribution and changes during pathological conditions. *CKD* chronic kidney disease, *COPD* chronic obstructive pulmonary disease

Blood vitamin C levels are lower in men than in women on average [39]. Reasons for this include the fact that men have larger bodies and a higher percentage of smokers. Notably, lower blood vitamin C levels have been observed in middle-aged and older adults than in younger adults in both men and women [40]. One reason for this is that older adults are more likely to be prone to chronic diseases. Therefore, vitamin C levels are closely associated with aging and diseases (Fig. 1).

In our laboratory, plasma vitamin C levels in patients with chronic kidney disease (CKD) were analyzed [7]. It was revealed that elderly patients with CKD had lower plasma vitamin C levels (27.1 ± 13.9 µM) than healthy elderly individuals (42.3 ± 15.5 µM). In particular, patients on hemodialysis had a higher risk of developing scurvy because the plasma vitamin C levels were 7.2 ± 3.9 µM after the hemodialysis, and the proportion of DHA increased. This may be due to the removal of vitamin C from the blood by hemodialysis and unbalanced nutritional intake due to dietary therapy. Some patients with diabetes have low blood and urine vitamin C levels due to poor vitamin C reabsorption or renal leakage caused by renal impairment [41]. In contrast, vitamin C supplementation may improve glycemic control and blood pressure in patients with type 2 diabetes [42].

Furthermore, our previous study has revealed that plasma vitamin C levels were lower in patients with chronic obstructive pulmonary disease (COPD) (31.2 ± 13.9 µM) [8]. A recent meta-analysis [43] showed that patients with COPD who received 400 mg of vitamin C daily had significantly improved respiratory function (forced expiratory volume in one second [FEV1%] and FEV1/forced vital capacity [FVC]) compared to the placebo group. In addition, a prospective cohort study in Europe [44] examined the association between blood vitamin C levels and respiratory diseases. The results showed that in a population with low (mean 28 µM [3–41 µM]) and high (mean 79 µM [66–242 µM]) blood vitamin C levels, the group with higher levels had a significantly lower risk of developing lung cancer and pneumonia. Nevertheless, in a U.S. study involving men aged 50 years and older who received either 500 mg/day of vitamin C or placebo and were followed up for an average of 8 years to assess cancer incidence [45], it was found that vitamin C intake had no effect on the incidence of lung cancer. Furthermore, an analysis showed no evidence of a causal association between circulating vitamin C levels and the risk of lung, breast, prostate, colon, and rectal cancers [46].

In a study of patients with cancer, low plasma vitamin C levels were more prevalent in patients undergoing cancer chemotherapy or immunotherapy than in pre-surgery patients [47]. In other words, cancer treatment likely affects blood vitamin C levels. Currently, no clear efficacy of high-dose intravenous vitamin C therapy has been demonstrated for cancer [48]. However, vitamin C may be effective against several types of cancer, and clinical trials are ongoing worldwide.

Blood vitamin C levels are also reduced by respiratory tract infections such as coronavirus infections and sepsis [49, 50]. These levels tend to be lower in critically ill patients [49, 50]. Vitamin C may have immunomodulatory functions, such as the suppression of pro-inflammatory cytokines such as interleukin (IL)-6, tumor necrosis factor (TNF)-α, and C-reactive protein (CRP), and reduce oxidative stress, which are increased during infection [51]. However, the therapeutic efficacy of vitamin C has been inconsistent among clinical trials and ineffective in large-scale studies [52, 53]. The effect of vitamin C on infections may be influenced by the baseline blood vitamin C levels in the individual [54].

Significantly increased odds of coronary artery disease were also observed in individuals with deficient plasma vitamin C levels [55]. It is plausible that chronic insufficiency of vitamin C, due to both inadequate nutritional intake and increased consumption in the body, contributes to the progression of chronic diseases involving inflammatory components.

There is also a significant decrease in plasma vitamin C levels in patients with Alzheimer’s disease (AD) compared to healthy controls [56]. However, no association has been reported between vitamin C levels and cognitive dysfunction in AD [57]. Notably, higher blood vitamin C levels are associated with a reduction in apolipoprotein E (APOE) E4-related risks of cognitive decline in women [58]. The APOE gene ɛ4 is a strong genetic risk for AD. Further studies are warranted to elucidate the exact mechanistic role of vitamin C in the pathophysiology and prevention of AD.

It has also been reported that middle- and older-aged men and women with lower plasma vitamin C levels (< 50 µM) have lower estimated skeletal muscle mass than those with high levels (≥ 50 µM) [59]. Our study showed a decrease in skeletal muscle weight due to long-term vitamin C deficiency in SMP30/GNL-KO mice, and this loss of muscle weight was reversed by vitamin C administration [30]. The amount of ROS contributes to protein degradation in skeletal muscles and vitamin C may affect it [29]. In addition, a cross-sectional study of elderly women (70–84 years old) showed a positive correlation between blood vitamin C levels, muscle strength, and physical performance [60]. Similarly, a negative correlation of blood vitamin C concentration with frailty severity has been reported in elderly individuals (≥ 75 years old) [61]. Muscle decline is one of the most significant causes of frailty, and maintaining high blood vitamin C levels may play a role in its prevention.

Moreover, a meta-analysis showed that a higher vitamin C intake was associated with a lower risk of osteoporosis [62]. Vitamin C deficiency is thought to cause collagen loss, leading to a decreased bone mineral density. In contrast, recent studies using *Gulo*-KO mice have shown that the epigenetic functions of vitamin C are central to osteoblastogenesis and bone formation [63]. Epigenetic abnormalities are associated with the development of many diseases [64], and the epigenomic modulatory effects of vitamin C are promising [65]. Interestingly, vitamin C intake has been associated with systemic development and the immune response by epigenetic regulation [66], and may have an effect on epigenetic age, an indicator of biological age.

Although the results of these studies varied depending on the dosage and duration of the study, the diverse physiological effects of vitamin C have been linked to various diseases. As a common cause, vitamin C levels are thought to be affected mainly by oxidative stress and acute or chronic inflammatory reactions (Fig. 1). However, the cause of low blood vitamin C levels may differ depending on the type of disease. Further mechanistic studies on the in vivo effects of vitamin C are required.

## Conclusions

Lower blood vitamin C levels have been observed in various pathological conditions and older adults. Therefore, maintaining a sufficient level of vitamin C may be beneficial for the prevention of age-related diseases and may be the key to longevity.

## Data Availability

Not applicable.

## Abbreviations

AD: Alzheimer’s disease
CKD: Chronic kidney disease
COPD: Chronic obstructive pulmonary disease
DHA: Dehydroascorbic acid
GNL: Gluconolactonase
Gulo: L-Gulono-γ-lactone oxidase
IL-6: Interleukin-6
ODS: Osteogenic disorder Shionogi
ROS: Reactive oxygen species
SMP30: Senescence marker protein-30
TNF-α: Tumor necrosis factor-alpha
